# Supplementary material for: Development of chemokine network inhibitors using combinatorial saturation mutagenesis
Source: Commun Biol. 2025 Apr 3;8:549. doi: 10.1038/s42003-025-07778-6 (PMC11969024; doi:10.1038/s42003-025-07778-6)
Supplement: Supplementary file 4 — Supplementary Data 2 [file 42003_2025_7778_MOESM4_ESM.zip › SupplementaryData3/scripts/xattune_dr.tar!/./script/analysis/JK/JK_E236_RA_THP1_plot.pdf]

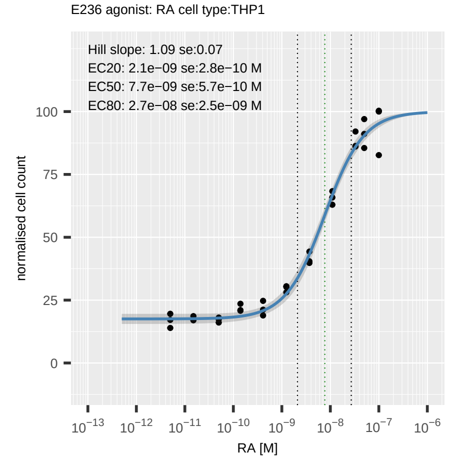

E236 agonist: RA cell type:THP1
Hill slope: 1.09 se:0.07
EC20: 2.1e−09 se:2.8e−10 M
EC50: 7.7e−09 se:5.7e−10 M
EC80: 2.7e−08 se:2.5e−09 M
normalised cell count
100
75
50
25
0
10^{-13}
10^{-12}
10^{-11}
10^{-10}
10^{-9}
10^{-8}
10^{-7}
10^{-6}
RA [M]
